# MALDI-TOF-MS for rapid screening analysis of M-protein in serum

**DOI:** 10.3389/fonc.2022.1073479

**Published:** 2022-12-15

**Authors:** Jie Li, Anping Xu, Weijie Xie, Bowen Li, Cunliang Yan, Yong Xia, Chao Liang, Ling Ji

**Affiliations:** ^1^ Department of Laboratory Medicine, Peking University Shenzhen Hospital, Shenzhen, China; ^2^ Department of Biology, School of Life Sciences, Southern University of Science and Technology, Shenzhen, China; ^3^ Institute of Integrated Bioinfomedicine and Translational Science (IBTS), School of Chinese Medicine, Hong Kong Baptist University, Hong Kong, Hong Kong SAR, China; ^4^ State Key Laboratory of Proteomics, National Center for Protein Science (Beijing), Beijing Institute of Lifeomics, Beijing, China

**Keywords:** plasma cell dyscrasias, serum, M-protein, MALDI-TOF-MS, screening test

## Abstract

Monoclonal immunoglobin (M-protein) is a serum biomarker for the diagnosis of plasma cell dyscrasias. Despite limitation of analytical sensitivity and resolution, serum protein electrophoresis and immunofixation electrophoresis are still the front-line tests for the detection of M-proteins. Herein, we developed a MALDI-TOF Mass spectrometry-based method for the screening test of M-proteins in human serum. Based on the unique mass signature of different immunoglobin isotypes, M-Proteins could be rapidly identified and typed. The method demonstrated with high analytical performance and throughput, rapid and simple, which could be a new choice for the diagnosis of plasma cell dyscrasias.

## 1 Introduction

Plasma cell dyscrasias (PCD) encompass a broad spectrum of diseases ranging from asymptomatic monoclonal gammopathy of undetermined significance (MGUS) to life-threatening diseases such as multiple myeloma, Waldenström’s macroglobulinemia and amyloid light chain (AL) amyloidosis (1–3). PCD are characterized by clonal expansion of plasma cells which can secrete monoclonal immunoglobin component (M-protein) (4). Detection of M-protein are integral parts in the diagnosis and post-treatment monitoring of PCD (5).

M-proteins are consisting of intact immunoglobin with both heavy and light chains, or with light/heavy chains only (i.e. with the diseases of light chain myeloma, AL amyloidosis, and heavy chain disease) (6–8). Front-line assays for the detection of M-proteins are mainly based on electrophoresis technologies. Serum protein electrophoresis (SPE) conducted by agarose gel or capillary zone electrophoresis is commonly used for the detecting and screening of M-proteins (9–11), while immunofixation electrophoresis (IFE) is classically used for ascertaining the presence and determining the isotypes of M-proteins (12). In addition, serum free light chain (sFLC) assay is another widely used method for quantification of circulating free kappa (κ) and lambda (λ) light chains (LCs) (13). However, these methods are either less sensitive or laborious. The application of newly monoclonal therapeutic antibodies (i.e. Daratumumab) also brought analytical interference to the M-protein detection when using electrophoretic methods (14, 15). Thus, novel methods with higher sensitivity and anti-interference ability are desired for the M-protein analysis.

Mass spectrometry-based methods have been developed for M-protein detection and PCD diagnosis in recent years (16, 17). Enhanced sensitivity has been demonstrated in previous studies. For instance, the clonotypic peptide mass spectrometry method with a bottom-up proteomic approach showed about 2000 times higher sensitivity than SPE (18). Requiring advanced bioinformatic and computational algorithms analysis lead to long turn-around time. Alternatively, top-down approach such as miRAMM (monoclonal immunoglobulin rapid accurate molecular mass) was developed based on using the accurate mass of the intact light chains as the detection marker of PCD (19). Although the sample preparation and data analysis are much simpler than clonotypic peptide mass spectrometry method, the workflow of miRAMM included liquid chromatography separation that made it still complex for common clinical laboratories (20). Most recently, chromatography-free system was developed by using MALDI-TOF mass spectrometry for the determination of M-proteins. Novel methods such as MASS-SCREEN and MASS-Fix were developed and validated in a few clinical centers (21, 22). However, these methods are based on immunoenrichment by using different types of nanobodies (NBs) that not only introduced time-consuming for sample incubation with NBs but also increased the cost of the testing. Besides, studies also demonstrated that nonspecific absorption of albumin onto the NBs brought analysis interference to the light chains mass region (21). Here, we developed a novel immunoenrichment-free MALDI-TOF-MS-based method (MDT-MALDI) for M-proteins detection and type confirmation. The assay successfully reduced the analytical interference of albumin in M-protein detection by using MALDI-TOF-MS. Meanwhile, without the usage of antibodies, the consuming time and cost are largely reduced. The current study provided a new choice which may facilitate for the screening test and disease monitoring of plasma cell dyscrasias.

## 2 Material and methods

### 2.1 Materials and reagents

Human serum IgG-, IgA- and IgM-standard (reagent grade, purity ≥95% by HPLC) were all from Sigma-Aldrich (US). All the standards are polyclonal. Certificated reference human serum (ERM-DA470K/IFCC) was obtained from Institute for Reference Materials and Measurements (IRMM, European Commission-Joint Research Center). Sinapinic acid (SA), α-Cyano-4-hydroxycinnamic acid (CHCA) and 2,5-Dihydroxybenzoic acid (DHB) matrix were obtained from Sigma-Aldrich (US). Albumin Depletion Kit (Thermo Fisher, US) was used for removing albumin proteins from serum samples.

### 2.2 Patients selection and serum sample collection

All the patient serum samples and data were assessed in compliance with the Institutional Medical and Ethics Committee of Peking University Shenzhen Hospital. Waste serum samples which previously tested by SPE and IFE in the Clinical Laboratory of Peking University Shenzhen Hospital were enrolled in the present study to evaluate the analytical sensitivity, specificity and limit of detection (LOD) of MDT-MALDI. Totally, 212 samples including 110 electrophoresis positive samples (62 SPE positive M-proteins and 48 M-proteins detectable by IFE only) and 102 IFE negative serum samples. In addition, the utility of current MALDI-TOF MS-based method for M-protein level monitoring was tested in a cohort of eight MM patients whose serum samples including the diagnostic and plus 5 available posttreatment samples from our biobank.

### 2.3 Sample preparation and mass spectrometry analysis

Albumin protein was removed from the serum samples using an Albumin depletion kit (Thermo Scientific). 10 μl of samples were incubated with 10 μl of 0.5 mol/L dithiothreitol (DTT) for 20 mins to disassociate immunoglobins into separated light chain and heavy chain components. Then mixed 1:9 with 20 mg/mL DHB matrix and spotted onto a stainless steel MALDI target plate (6×16 sample array) using the modified sandwich matrix application method (23). 0.5 μl of the matrix DHB was pre-spotted onto the plate, additional 2 μl of the sample-matrix mixture were added to the spot and allowed to dry. Simultaneously, a series of human serum IgG-, IgA- and IgM-standard samples (Sigma- Aldrich) were prepared in the same manner to establish the reference spectra on each target plate.

All spectra were acquired on QuanTOF (Intelligene Biosystems, China) with the following settings: source voltage 19 kV, laser frequency 5 kHz, laser energy 15 μJ, scanning speed 2 mm/s, mass range 5000–30,000 m/z, 15-rows scan per spot. Mass spectrometric analysis speed by QuanTOF was about 15s per sample spot with the above settings. Mass spectra were processed by QuanTOF viewer software developed by Intelligene Biosystems for QuanTOF instrument.

### 2.4 Serum protein electrophoresis (SPE) and immunofixation electrophoresis (IFE) analysis

SPE was performed on a CAPILLARYS 2 instrument (Sebia, Lisses, France) according to the manufacturer’s instructions. SPE is considered positive when the M-protein is confirmed by IFE and there is a distinct restriction (band) within the polyclonal background. IFE was carried out using Hydrasys 9 IF gels (Sebia, Lisses, France) and incubated with anti-sera for heavy chain (γ, α, and μ) and light chain (κ and λ), respectively. Acid violet was used for the gel staining. If a light chain with no corresponding γ, α, and μ heavy chain was detected, the sample was retested with anti-sera for δ and ϵ heavy chain.

### 2.5 sFLC analysis and other biochemical index

Serum free light chain κ and λ were determined by FreeLite™ reagents (Binding Site, UK) on a Protein Chemistry Analyzer IMMAGE800 (Beckman Coulter, USA). In addition, the total protein concentration and other biochemical index were measured by colorimetric assay on a Clinical Chemistry Analyzer AU5831 (Beckman counter, USA).

## 3 Result

### 3.1 Method and workflow establishment of MDT-MALDI

**Figure 1:**
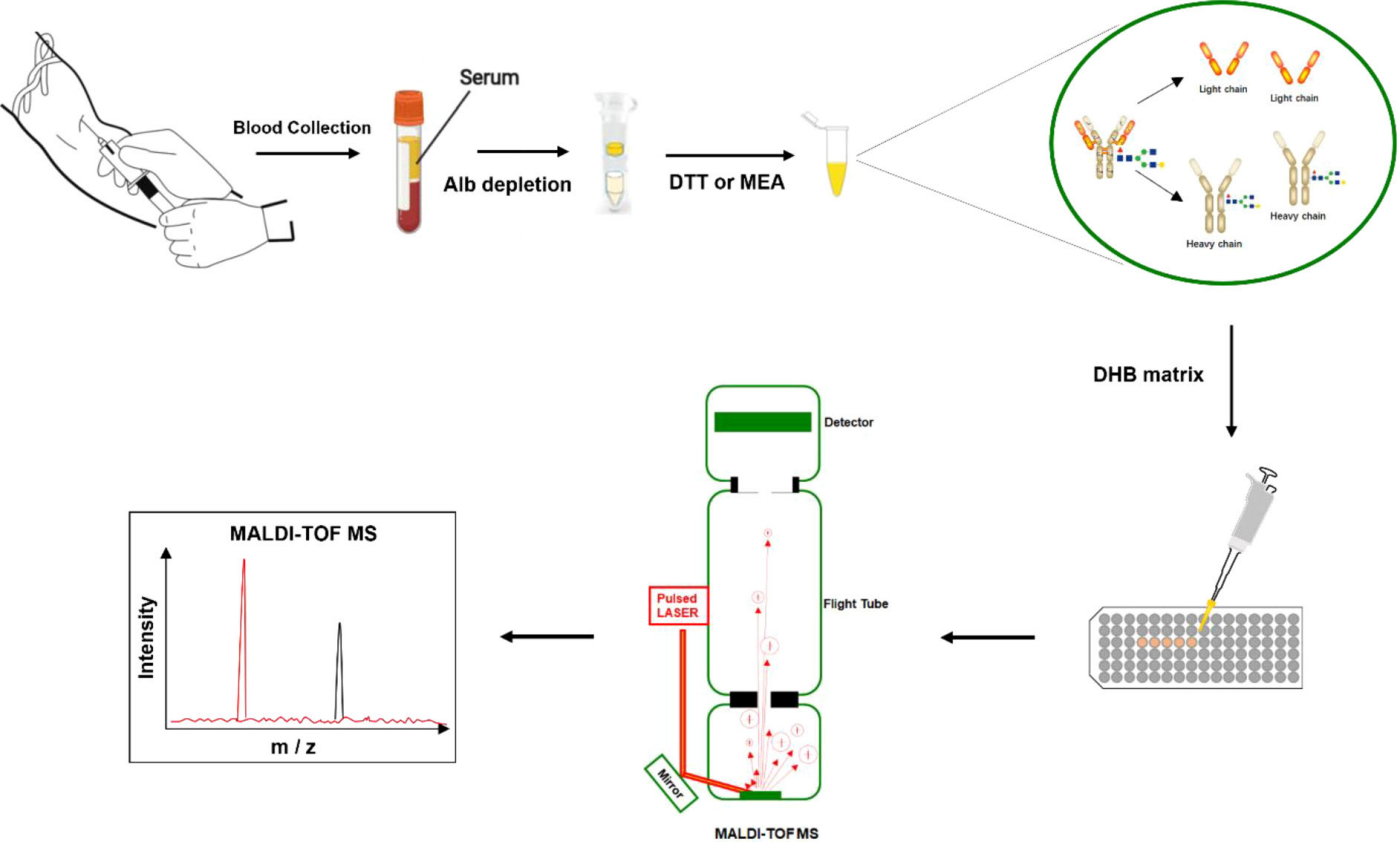
Workflow diagram of the MALDI-TOF MS method for the screening test of M- protein in serum.

**Figure 2:**
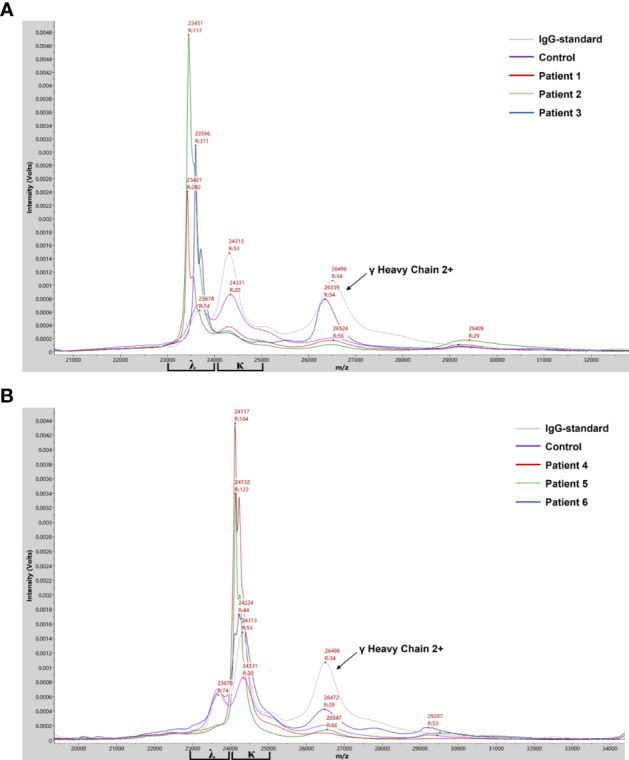
Mass spectra from patient serum samples revealed the clonal λ **(A)** or κ **(B)** light chain distribution of the M-proteins. Mass spectra from serum samples of IgG standard, health control and M-protein positive patients with λ- (patient 1-3) or κ- light chain (patient 4-6) were in different colors. Arrows indicated peaks correlated to the [M+2H]^2+^ of λ heavy chain.

**Figure 3:**
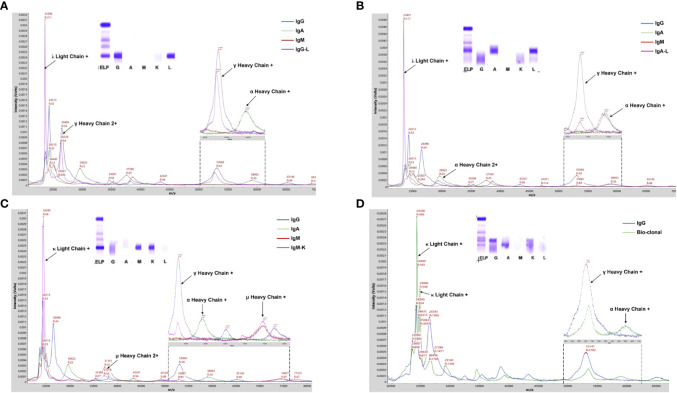
MALDI-TOF MS analysis of serum samples from PCD patients. **(A-C)** Mass spectra from patient serum samples with IgG-L, IgA- Land IgM-K M-proteins showed typical γ, α, and u heavy chains peaks, respectively. **(D)** Mass spectra from a patient with bi-clonal gammopathies reveled two types of M-proteins including IgG-K and IgA-K. Arrows indicated peaks correlated to the corresponding component of Ig molecules. Mass spectra from IgG/A/M standard and patient serum samples were in different colors.

The protocol for M-protein determining and type confirmation by MALDI-TOF MS (MDT-MALDI) was optimized and established (**Supplementary Materials**). Detailed workflow of the assay is illustrated in **Figure 1**. The light chain (κ and λ) molecular mass distributions observed using in current method were similar to previous studies (21, 22). Serum samples with M-proteins were identified as sharp peaks which can be distinguished from the polyclonal background referring to the normal health control and standard serum samples (**Figure 2**). Moreover, the heavy chain types of M-proteins can be identified by the intensity and the characteristically peaks of [M+H]^+^ and [M+2H]^2+^ of HC component as comparing with the overlaid mass spectrums of the IgG/A/M standards (**Figures 3A–C**). Furthermore, mass spectra from the patient with bi-clonal gammopathies revealed two types of M-proteins by using the current method (**Figure 3D**).

### 3.2 Sensitivity, specificity and LOD evaluation of MDT-MALDI

**Figure 4:**
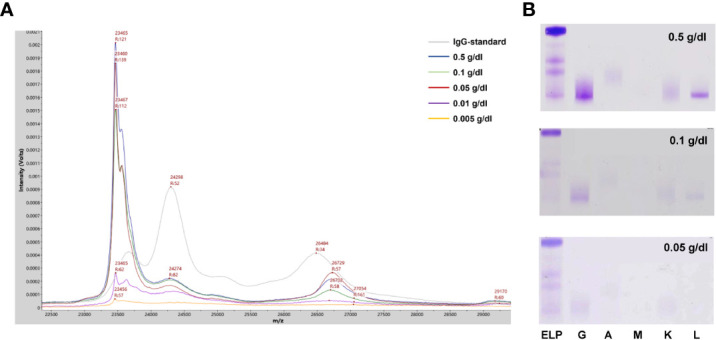
LOD analysis of the MALDI-TOF MS method. **(A)** Mass spectra from a IgG-L multiple myeloma patitent serum sample with series dilution (0.005-0.5 g/dl). **(B)** Parallel testing of the serum sample with immunofixation electrophoresis.

To evaluate the efficiency of the current method for M-proteins’ detection, a cohort of 212 patients’ sera previously tested with SPE and IFE was used for the method comparison. All the 62 patients with SPE positive results could be identified by the current MALDI-TOF MS method. In contrast, additional 43 SPE-/IFE+ and 7 cases with SPE-/IFE- patient were detected as positive with our method, indicated a higher analytical sensitivity. Compared to the IFE positive/negative results, the overall sensitivity and specificity of the MDT-MALDI assay were determined as 95% and 93%, respectively. Among the different isotypes of M-proteins, the consensus was reached as 93.8% to 100% (**Table 1**). To further assess LOD of the current method, 5 different SPE positive M-protein samples were subsequently diluted into series concentrations and detected by IFE and MDT-MALDI, respectively. As indicated in **Figure 4A**, the LOD of the current method was between 0.005 to 0.01 g/dl. In addition, the LOD evaluation was also tested by using human standard serum (ERM-DA470K/IFCC) with series dilution. Similar trend was also found in the standard serum samples (**Supplementary Figure S3**). The analytical sensitivity of MDT-MALDI was higher than IFE (**Figure 4B**). Furthermore, the time and cost consuming between the two methods was also evaluated. Based on the experience in our lab, at least 2 hours were needed for one gel with 9 samples using the IFE testing, while the entire analysis time of 96 samples including centrifuging, albumin depletion and analysis procedure was less than 3 hours using our developed approach. More importantly, the current method does not require antibodies, thus significantly reducing the cost as compared with IFE.

**Table 1:** Comparison between MDT-MALDI and SPE/IFE.

Test Method		SPE+/IFE+	SPE-/IFE+	SPE-/IFE-
MDT-MALDI Positive		62	43	7
MDT-MALDI Negative		0	5	95
Total		62	48	102
Specificity analysis by isotype				
**Isotype**	**N**	**MDT-MALDI +**	**MDT-MALDI -**	**Agreement**
IgG	53	51	2	96.20%
IgA	32	30	2	93.80%
IgM	18	17	1	94.40%
FLC	4	4	0	100%
Biclonal	3	3	0	100%
Negative	102	7	95	93.10%

SPE, Serum protein electrophoresis; IFE, immunofixation electrophoresis.

### 3.3 Utility analysis of MDT-MALDI in disease monitoring

**Figure 5:**
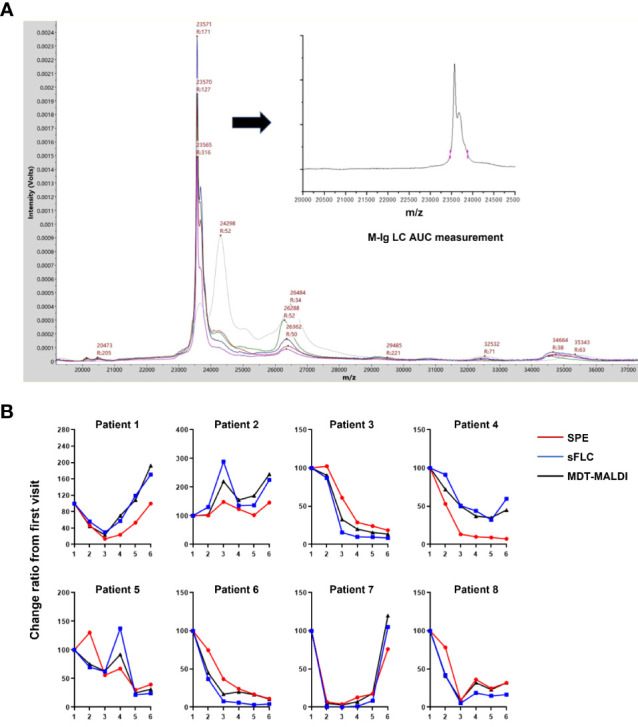
Utility analysis of MDT-MALDI in disease monitoring. **(A)** Mass spectra from the serial serum samples (diagnostic and following post-treatment) of an IgG-L multiple myeloma patient. M-protein concentration change between series samples can be calculated by the AUC measurement for gated LC signal and comparing with the first visit time for disease monitoring. **(B)** Comparison of the change ratio of M-protein concentrations determined by SPE, sFLC assay and MDT-MALDI. For each assay, the M-protein concentration (M-Ig AUC) at diagnosis was set at 100% and the relative M- protein concentration was calculated for each serial sample. A cohort of 8 patients with a history of multiple myeloma and disease monitoring serum samples were tested.

To further evaluate the utility of MDT-MALDI in monitoring M-protein levels, a cohort of 8 multiple myeloma patients whose serum samples including the diagnostic and plus 5 post-treatment sample were tested. The cohort included 6 IgG and 2 IgA multiple myeloma patients that had SPE, IFE and sFLC results available for each sample. All the serum samples were subsequently tested with MDT-MALDI. The relative change in M-protein concentration from the diagnostic sample was calculated by the AUC measurement of the monoclonal LC signal (22). Among these patients, the relative change ratio in M-protein concentration in a time-series samples were plotted for SPE, sFLC and MDT-MALDI, respectively (**Figure 5**). Person correlation coefficient analysis revealed that MDT-MALDI exerted higher agreement score with sFLC assay than SPE (**Supplementary Table S1**). Particularly, Patient 4 was noted to have clinical manifestations indicating progressive disease during visit 5 to 6. Both sFLC and the MDT-MALDI assay demonstrated a quantitative increase in disease burden that related to the disease progression earlier than SPE (**Figure 5B**).

## Discussion

The current study developed a novel immunoenrichment-free MALDI-TOF-MS-based method that could detect and isotypic identification of M-protein from serum samples in one assay. One of the major technical improvements that achieved in present study is reducing the analytical interference of serum albumin for M-protein analysis. As the most abundant proteins in serum, nonspecific absorption of albumin onto the NBs brought analysis interference in the determination of light chains mass region have been noticed in previous studies (21). Although the present study used an immunoenrichment-free method for M-protein analysis, [M+3H]^3+^ of albumin with high intensity still showed analytical interference with the light chain (particularly λ chain) component of immunoglobins. After optimizing the procedure, not only the confluent peak of albumin with lambda light chain diminished, but also the signal intensity of light chains and heavy chains of M-proteins were significantly enhanced after the albumin removal.

Another technical improvement in the current study is to optimize the most suitable matrix for M-protein analysis. Sinapinic acid (SA), 2,5-dihydroxybenzoic acid (DHB) and α-cynao-4-hydroxycinnamic acid (CHCA) are commonly used matrix for MALDI-TOF MS analysis. Particularly, CHCA has been successfully used for the M-proteins analysis in other studies (21, 22). However, mass spectra from those studies showed that the signal intensity of [M+2H]^2+^ (m/z: 11000~12500) of κ or λ light chain was higher than their corresponding [M+H]^+^ (m/z: 22000~25000) charge state in CHCA matrix. It indicated that CHCA may be suitable for smaller proteins analysis. The present study tested the above three commonly used matrix and found that DHB matrix is the optimum matrix for M-protein analysis. Similar with other studies (21, 22), the signal intensity of [M+2H]^2+^ of κ or λ light chain was higher than their corresponding [M+H]^+^ charge state in CHCA matrix, while low signal intensity of heavy chain component for both [M+H]^+^ and [M+2H]^2+^ charge states in CHCA matrix were observed in our study. On the contrary, the MS signal intensity of the light chain and especially heavy chain component were significantly enhanced in the DHB matrix. Studies has demonstrated that DHB matrix is preferred in protein posttranslational modification analysis including glycans and proteins with glycosylation modification (24). Since glycosylation is a commonly posttranslational modification in immunoglobulins (including M-proteins) particularly formed in the heavy chains (25, 26). This feature of M-protein may contribute to the signal enhancement of heavy chain component of M-protein in DHB matrix.

Limitations also need to be faced when using MDT-MALDI. Previous studies have reported that a little portion of patients (more likely with AL amyloidosis) with glycosylation in light chains may have abnormal peaks and with a mass outside the normal expected range in MALDI-TOF analysis (27, 28). For these patients, the present of M-protein could be screened by the atypical peaks, while the confirmation of types may need supplementary testing such as IFE. Besides, unlike the typical non-gaussian distribution of light chain signature (spike peaks), the heavy chain type determination was mainly based on the signal intensity corresponding to the relative m/z signatures, i.e. [M+H]^+^ and [M+2H]^2+^ of HC component. Bias maybe introduced by different viewer for the spectra analysis. Enlargement of sample size particularly atypical spectra for the training set are needed in the further blinded assessment studies. Meanwhile, additional quantification of IgG/A/M levels by clinical chemistry analyzer also can be taken into consideration for improving the screening specificity.

In summary, the current study developed a novel MALDI-TOF-MS-based method (MDT-MALDI) for the detection and isotypic identification of M-protein in serum samples. MDT-MALDI exerted higher agreement score with sFLC assay than SPE for disease monitoring. Compared to the front-line electrophoresis technologies, the current assay demonstrated with high analytical performance and throughput, more rapid, convenient and economical. The current method could be a new choice for the diagnosis and disease monitoring of plasma cell dyscrasias.

## Data availability statement

The original contributions presented in the study are included in the article/**Supplementary Material**. Further inquiries can be directed to the corresponding authors.

## Ethics statement

The studies involving human participants were reviewed and approved by Institutional Medical and Ethics Committee of Peking University Shenzhen Hospital. The patients/participants provided their written informed consent to participate in this study.

## Author contributions

JL, CL and LJ supervised and revised the manuscript. AX, WX and BL collected the clinical samples, JL conducted the main experiments. CY and YX provided the professional expertise. All authors contributed to the article and approved the submitted version.
