# Supplementary material for: MALDI-TOF-MS for rapid screening analysis of M-protein in serum
Source: Front Oncol. 2022 Dec 15;12:1073479. doi: 10.3389/fonc.2022.1073479 (PMC9797962; doi:10.3389/fonc.2022.1073479)
Supplement: Supplementary file 1 [file DataSheet_1.pdf]

Supplementary Material

Materials and reagents

Human serum IgG-, IgA- and IgM-standard (Cat#I2511, I4036 and I8260, reagent grade, purity ⩾95% by HPLC) were all from Sigma-Aldrich (US). Sinapinic acid (SA:10mg/mL in ACN:0.1%TFA, 3:7), α -Cyano-4-hydroxycinnamic acid (CHCA: 10mg/mL in ACN:0.1% TFA, 7:3) and 2,5-Dihydroxybenzoic acid (DHB: 20mg/mL ACN:0.1%TFA, 1:1) matrix were used for matrix selection. Albumin Depletion Kit (Thermo Fisher, US) was used for removing albumin proteins from serum samples.

Sample preparation

Ten microliters of samples were incubated with 10μl of 0.5mol/L dithiothreitol (DTT) for 20 mins to disassociate immunoglobins into separated light chain and heavy chain components. Then mixed 1:9 with CHCA, SA or DHB matrix and spotted onto a stainless steel MALDI target plate (6×16 sample array) using the modified sandwich matrix application method [23]. A half microliter of the matrix was prespotted onto the plate, additional two microliters of the sample-matrix mixture were added to the spot and allowed to dry.

MALDI-TOF MS analysis

All spectra were acquired on QuanTOF (Intelligene Biosystems, China) with the following settings: source voltage 19 kV, laser frequency 5 kHz, laser energy 15μJ, scanning speed 2 mm/s, mass range 5000–30,000 m/z, 15-rows scan per spot. Mass spectrometric analysis speed by QuanTOF was about 15 s per sample spot with the above settings. Mass spectra were processed by QuanTOF viewer software developed by Intelligene Biosystems for QuanTOF instrument.

MDT-MALDI method establishment

M-protein is derived from recombination and somatic hypermutation events of both the heavy- and/or light-chain loci of the clonal plasma cells [1]. Although each M-protein has both a unique amino acid sequence and unique molecular mass, it still can be categorized into the five types (IgG/A/M/D/E) of immunoglobulins or free light chains (κ/λ) only. The basic structure of all immunoglobulin molecules is a unit consisting of two identical light chains (MW: 22000~25000) and heavy chains (MW: 50000~77000). Mass spectrometry is ideally suited for accurate mass measurement and distinguishing between the Ig classes. In the present study, human serum IgG-, IgA- and IgM-standard which diluted in DI water (0.01~1 mg/ml) were used for the method establishment and protocol optimization.

To find out the best matrix for the method establishment, the most commonly used matrix (SA, CHCA and DHB) for protein and peptide sample ionization were tested. The Ig light chains (κ and λ) molecular mass distributions were similar to previous studies [2]. Light chain signals (MW: 22000~25000) were obtained with higher intensity when using the SA and DHB matrix (Supplementary Figure 1A-B). All the heavy chain signals of Ig (50000~77000) were present with higher intensity when using the DHB matrix (Supplementary Figure 1A-C). More importantly, the γ, α, and μ heavy chains corresponding

to IgG/A/M component could be clearly distinguished based on the mass size different in the mass spectra by using DHB matrix (Supplementary Figure 1D).

As the protocol for detection and isotypic identification of immunoglobulins was well established. Serum samples from normal control patients (without M-proteins) were analyzed by MALDI-TOF MS for the immunoglobins detection. Supplementary Figure 2A displayed the typical spectrum that present with a polyclonal background of light chains of immunoglobulins from the normal patients. However, the $[M+3H]3+$ of albumin with high intensity exerted analytical interference with the light chain (particularly λ chain) component of immunoglobins. After optimizing the procedure using an albumin depletion kit, the confluent peak of albumin with lambda light chain diminished (Supplementary Figure 2B). In addition, both the signal intensity of light chains and heavy chains were significantly enhanced after the albumin removal (Supplementary Figure 2B). Thus, the albumin depletion procedure was adopted in the sample preparation procedure for MALDI-TOF MS analysis. The ability of current established method for M-protein detection were further analyzed and reported in the main text.

Supplementary Reference

1. Willrich MAV, Murray DL, Kyle RA. Laboratory testing for monoclonal gammopathies: Focus on monoclonal gammopathy of undetermined significance and smoldering multiple myeloma. Clin Biochem 2018;51:38-47.
2. Kohlhagen MC, Barnidge DR, Mills JR, et al. Screening Method for M-Proteins in Serum Using Nanobody Enrichment Coupled to MALDI-TOF Mass Spectrometry. Clin Chem 2016;62:1345-52.

Supplementary Figures

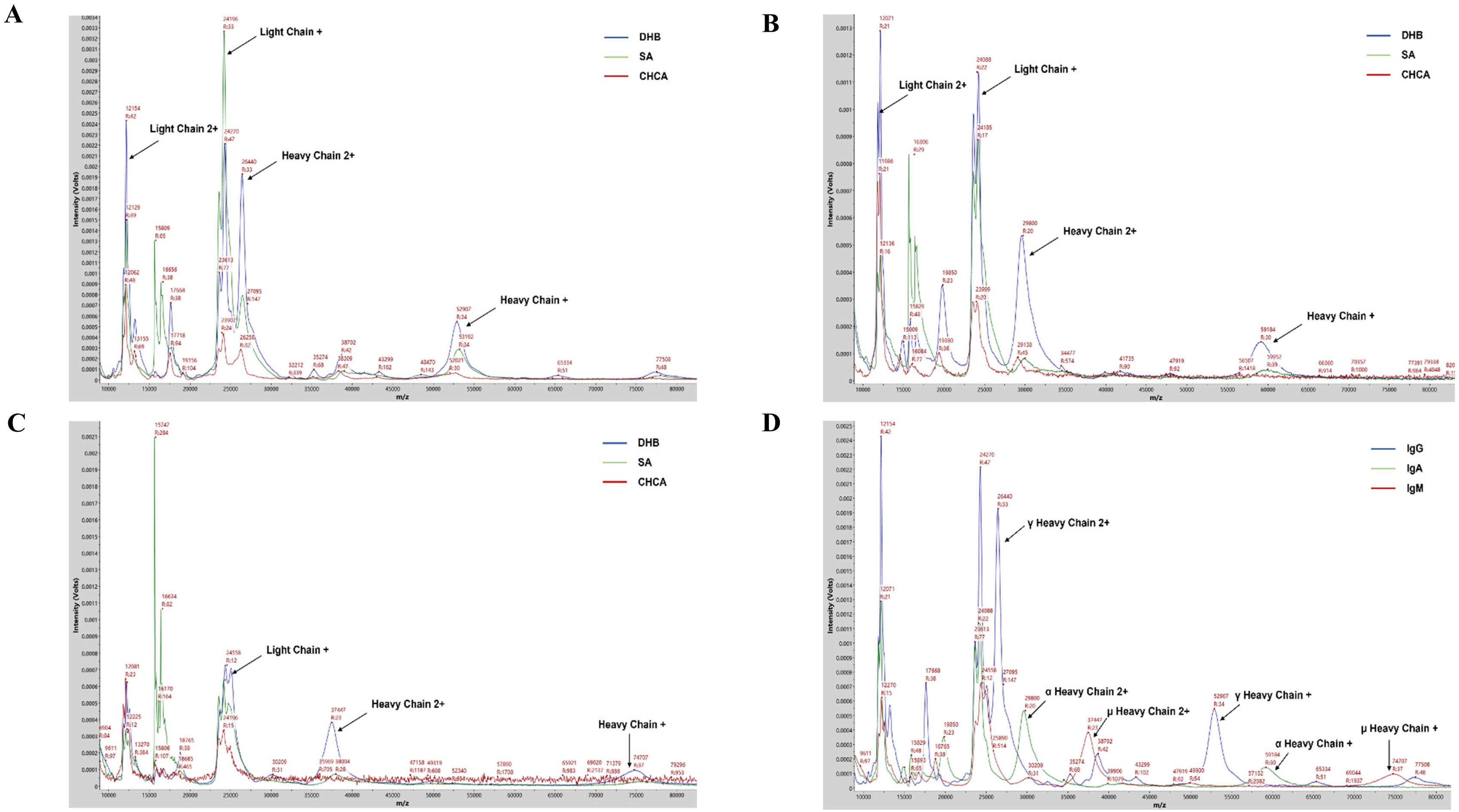

Supplementary Figure 1 MALDI-TOF MS analysis of human serum IgG-, IgA- and IgM-standard. (A-C) Serum IgG-, IgA- and IgM-standard analysis by MALDI-TOF MS using DHB, SA and CHCA matrix, respectively. **(D)** Mass spectra of the light chain (m/z 22000~25000) and heavy chain component (m/z 50000~77000) among the serum IgG-, IgA- and IgM-standard by using DHB matrix. The γ, α, and μ heavy chains corresponding to IgG/A/M could be clearly distinguished based on the mass size difference in the mass spectra. Arrows indicated peaks correlated to the corresponding component of Ig molecules.

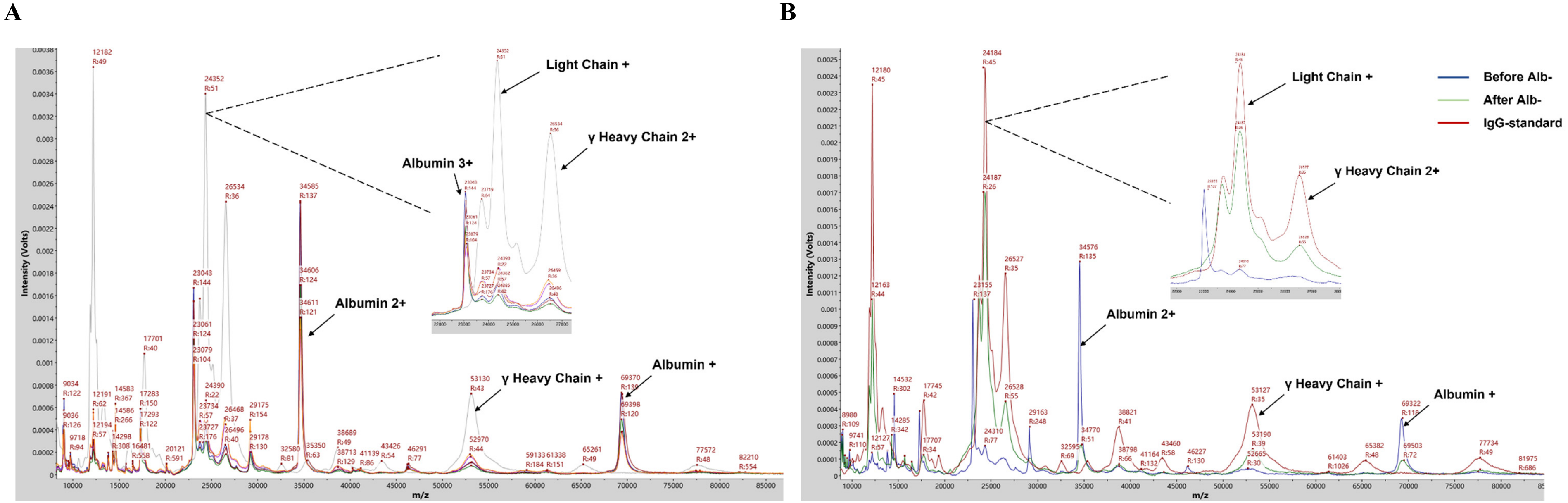

Supplementary Figure 2 MALDI-TOF MS analysis of Sera from healthy donors. (A) Polyclonal background of immunoglobins presented in human serum from healthy donors. Gray line indicated IgG standard serum. **(B)** Mass spectra comparison shown that albumin depletion reduced the interference from $[M+3H]^{3+}$ of albumin to the light chain component and enhanced the signal of heavy chain component in MALDI-TOF MS. Arrows indicated peaks correlated to the corresponding component of immunoglobins molecules and albumin. Expanded views of LC m/z distribution for the analysis of albumin interference.

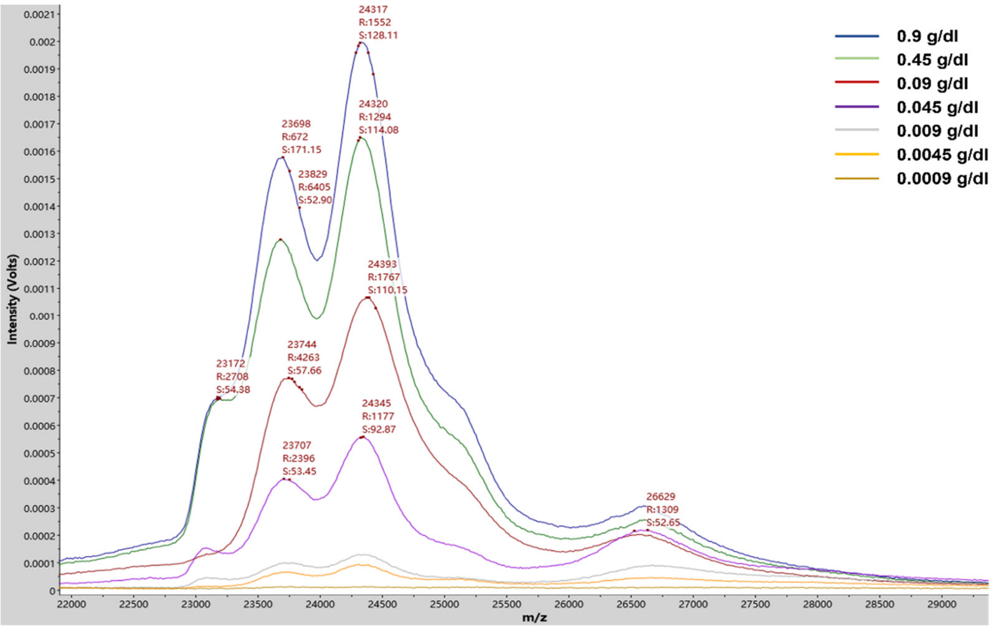

Supplementary Figure 3 LOD analysis of the standard serum by using MDT-MALDI. Mass spectra from human standard serum (ERM-DA470K/IFCC) samples with series dilution (0.0009~0.9g/dl).

Supplementary Table 1 Time-series Pearson correlation coefficients among the 8 patients

Patient#	MDT-MALDI vs sFLC	MDT-MALDI vs SPE	sFLC vs SPE
Patient 1	0.98	0.80	0.83
Patient 2	0.85	0.89	0.91
Patient 3	0.99	0.97	0.94
Patient 4	0.94	0.97	0.89
Patient 5	0.92	0.71	0.49
Patient 6	0.99	0.94	0.93
Patient 7	0.99	0.95	0.97
Patient 8	0.98	0.90	0.93
